# Surgical Strategy in Bouveret’s Syndrome: Report of a Case With One-Stage Surgery

**DOI:** 10.7759/cureus.56707

**Published:** 2024-03-22

**Authors:** Keiji Nagata, Takahisa Fujikawa

**Affiliations:** 1 Surgery, Kokura Memorial Hospital, Kitakyushu, JPN

**Keywords:** bailout procedure, subtotal cholecystectomy, one-stage surgery, gallstone ileus, bouveret’s syndrome

## Abstract

Bouveret’s syndrome is a rare condition caused by a gallstone that impacts the duodenum via a cholecystoduodenal fistula and obstructs the gastric outlet. Despite its high mortality rate, the treatment strategy for Bouveret’s syndrome is debatable and frequently challenging. The main issue is whether cholecystectomy and fistula repair following stone extraction should be performed concurrently with one-stage surgery. We present a case of Bouveret’s syndrome that was treated with one-stage surgery using a bailout procedure.

## Introduction

Bouveret’s syndrome is an uncommon complication of gallstone-related diseases, characterized by the obstruction of the gastric outlet. This obstruction occurs when a gallstone is impacted into the duodenum through a cholecystoduodenal fistula [1]. Persistent inflammation and adhesion between the gallbladder and the gastrointestinal tract increase the intraluminal pressure. Consequently, this causes wall ischemia and perforation, ultimately resulting in a cholecystoenteric fistula [2].

Gallstone ileus affects approximately 0.3-0.5% of individuals with cholelithiasis. Among these cases, Bouveret’s syndrome accounts for merely 1-3% [2,3]. Despite the progress in medical science, Bouveret’s syndrome still maintains a high mortality rate. This is mainly attributed to the characteristics of the afflicted patients, who are of advanced age and have multiple underlying health conditions and complicated cases with severe inflammation [4].

A variety of therapeutic procedures have been described in the treatment of Bouveret’s syndrome; however, whether cholecystectomy and fistula repair following stone extraction in the same operation as one-stage surgery should be performed or not remains debatable because one-stage surgery is considered to be overly invasive [5-8]. Here, we present a case of Bouveret’s syndrome treated with one-stage surgery.

## Case presentation

**Figure 1:**
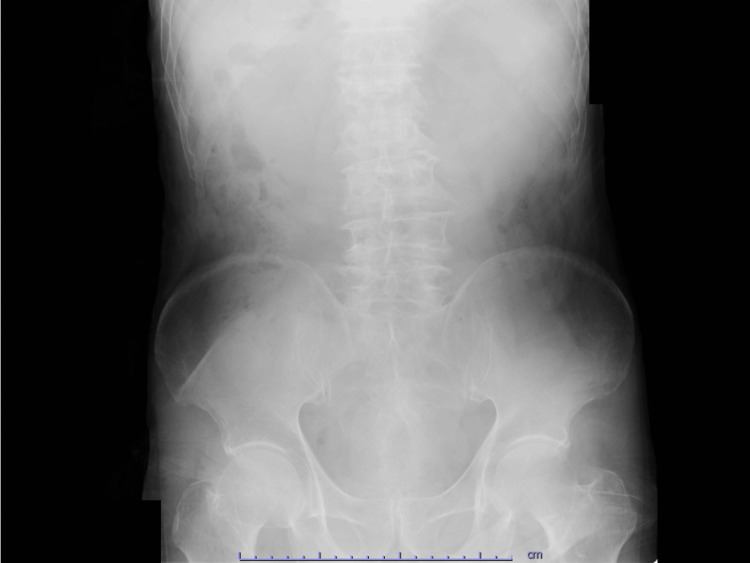
The abdominal X-ray on admission The abdominal X-ray on admission did not show signs of bowel obstruction, calcified density, and air in the biliary tract.

An 88-year-old Japanese man with a history of atrial fibrillation, hypertension, and Alzheimer's disease presented to the emergency department with complaints of abdominal pain, nausea, and persistent vomiting lasting throughout the night. He suffered from anorexia, general malaise, and prostration one week before arriving at the hospital. He took warfarin and antihypertensive drugs orally. He had no previous history of abdominal surgery or cholecystitis. He was hemodynamically stable and afebrile, but he had repeated episodes of gastric juice-like vomiting, as well as clinical signs of dehydration such as tachycardia and emaciation. On physical examination, his abdomen was soft with no tenderness, distention, or peritoneal symptoms. His laboratory values were unremarkable except for a mildly elevated C-reactive protein (CRP) level of 1.5 mg/dL, a prothrombin time (PT)-international normalized ratio (INR) level of 2.34, and an elevated alkaline phosphatase level of 453 IU/L. The liver function tests were within the normal limits. The abdominal X-ray revealed no signs of bowel obstruction, calcified density, or air in the biliary tract (Figure 1).

**Figure 2:**
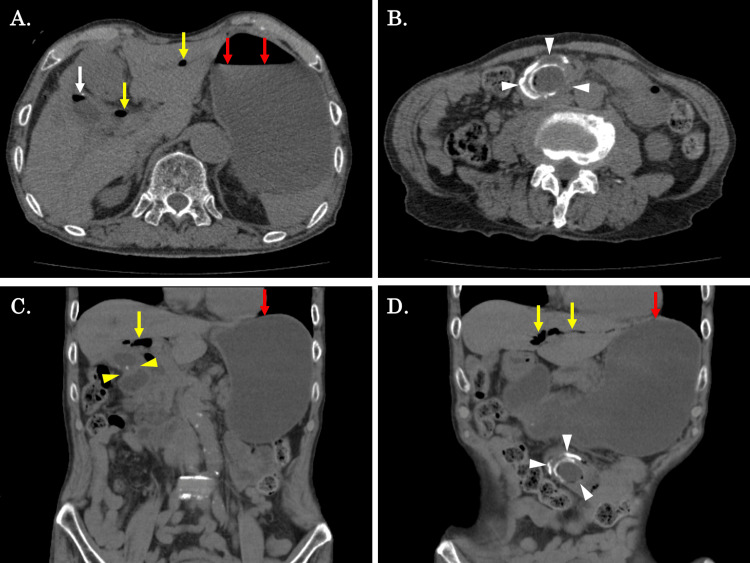
Abdominal CT findings on admission (A, B) An axial CT scan revealed air in the gallbladder (white arrows) and pneumobilia (yellow arrows). The stomach was dilated and filled with fluid (red arrows). A 4 x 3 cm calcified gallstone was located in the third portion of the duodenum (white arrowheads). (C, D) A coronal CT scan showed a cholecystoduodenal fistula (yellow arrowheads). A 4 x 3 cm calcified gallstone was located in the third part of the duodenum (white arrowheads), pneumobilia (yellow arrows), and stomach was dilated and filled with fluid (red arrows), but there was no small bowel dilatation distally.

Because he continued to vomit frequently even after arriving at the hospital, abdominal computed tomography (CT) was then performed. Abdominal CT revealed air in the gallbladder, pneumobilia, cholecystoduodenal fistula, and a calcified oval structure consistent with a gallstone in the third portion of the duodenum. The proximal duodenum and stomach were dilated and filled with fluid, but the small intestine was not dilated distally (Figure 2). This gallstone was assumed to have migrated via the fistula between the gallbladder and duodenum bulb, causing duodenum outlet obstruction.

**Figure 3:**
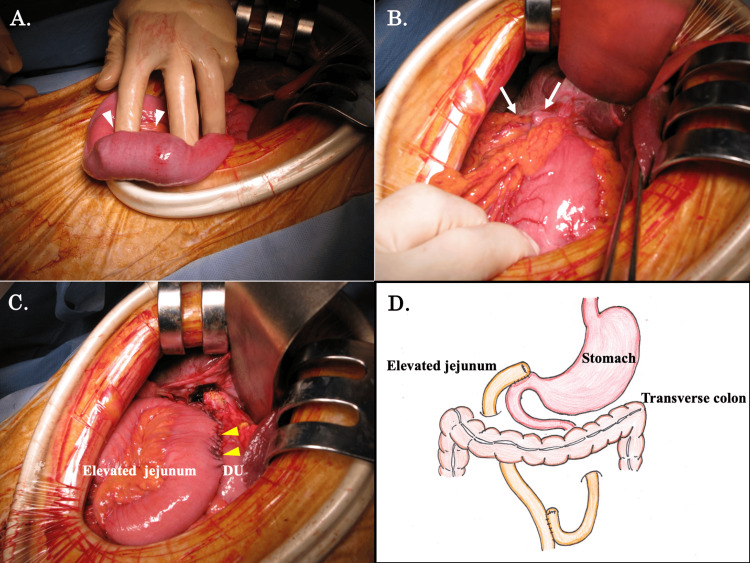
Operative findings (A) A gallstone is located in the upper jejunum, 30 cm from the Treatise ligament (white arrowheads). (B) The gallbladder was firmly adherent to the duodenal bulb (white arrows). (C) We elevated the resected jejunum on the anorectal side retrocolically and repaired the duodenal defect using the jejunal patch with an all-layer interrupted suture (yellow arrowheads). (D) Illustration of the surgical procedure. DU, duodenum Image Credits: Dr. Keiji Nagata

Bouveret’s syndrome was diagnosed based on the clinical presentation and CT findings. An emergency laparotomy surgery was performed because the gallstone was large and unlikely to migrate toward the anal side, and even if it drained, cholecystitis and cholangiotitis are expected to occur because of the cholecystoduodenal fistula. A gallstone was found in the upper jejunum, 30 cm from the Treitz ligament, rather than the third part of the duodenum (Figure 3A). The stone was thought to have migrated to the small intestine before surgery. No other gallstones were discovered. The gallbladder was collapsed and found to be firmly adhered to the duodenal bulb (Figure 3B).

**Figure 4:**
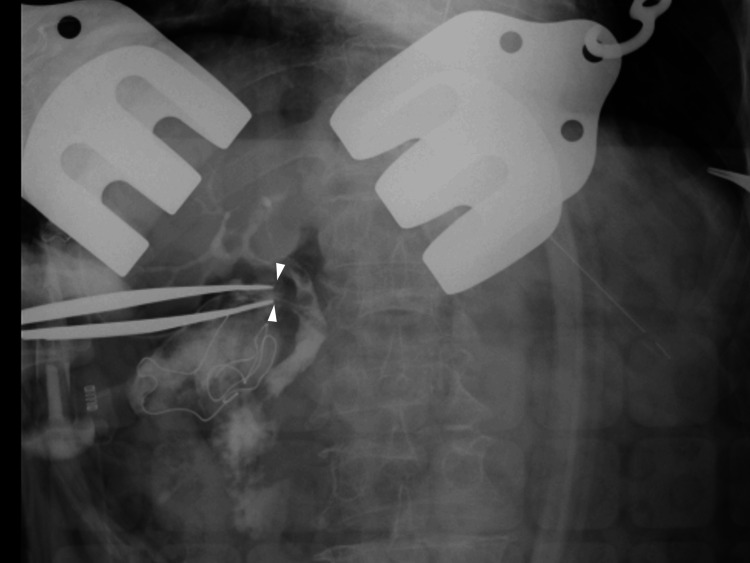
Intraoperative cholangiography Intraoperative cholangiography through the gallbladder lumen identified a cystic duct (white arrowheads).

**Figure 5:**
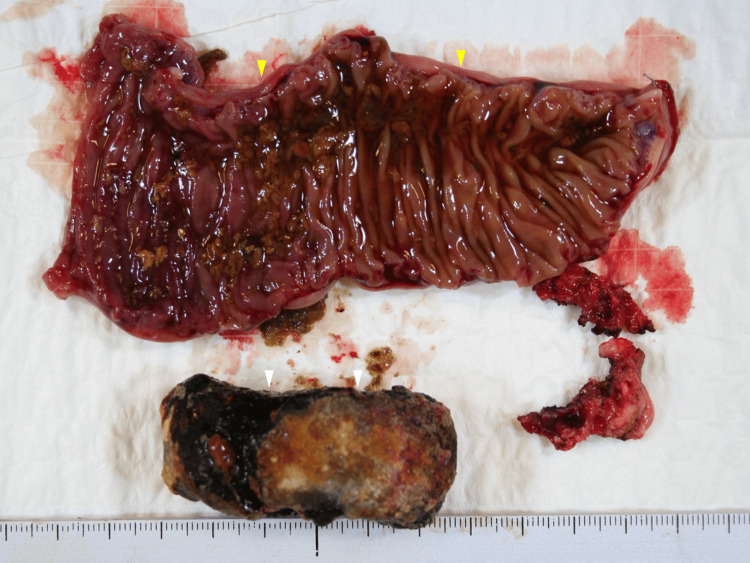
Macroscopic findings of the operative specimen The extracted gallstone, measuring 4x 3 cm (white arrows), and the upper jejunum (yellow arrows)

After incising the gallbladder wall, we identified the cholecystoduodenal fistula, which was about 3 cm in diameter, from the gallbladder lumen. As the inflammation surrounding the cholecystoduodenal fistula was extensive, we dissected the gallbladder from its fundus and performed a subtotal cholecystectomy. Intraoperative cholangiography was performed through the gallbladder lumen to identify the cystic duct (Figure 4), and the cystic duct orifice was sutured using a reconstituting technique. The duodenal wall and pyloric ring were stenotic and sclerotic, with thickened walls because of inflammation. To relieve the stenosis, we incised the anterior wall from the duodenal fistula toward the pylorus. We performed a partial jejunotomy to remove the gallstone lodged in the jejunum. We elevated the resected jejunum on the anorectal side retrocolically and repaired the duodenal defect using the jejunal patch with an all-layer interrupted suture (Figure 3C). We performed a Y-limb anastomosis 40 cm from the anastomotic site (Figure 3D). The postoperative course was uneventful, and the patient was discharged on the nineteenth postoperative day. An image of the resected jejunum and the gallstone is shown in Figure 5.

## Discussion

The symptoms of Bouveret’s syndrome are generally non-specific, most commonly featuring a triad of symptoms, including epigastric pain, nausea, and vomiting, which is presented as gastric outlet obstruction. Additionally, it may cause distension, upper gastrointestinal bleeding, fever, weight loss, and anorexia [9]. Given the non-specific symptoms of Bouveret’s syndrome, the diagnosis relies on clinical presentation and various imaging modalities. The classic triad of Rigler (signs of a distended stomach, calcified ectopic gallstone, and pneumobilia) on a plain abdominal X-ray is used to diagnose gallstone ileus [10], but it is not always useful. This triad appears on plain X-ray, the most common imaging modality, in only 14.8-21% of patients [2]. In recent years, CT has been performed relatively early in the emergency departments for abdominal discomfort, making it easier to diagnose these symptoms [11]. Cappell and Davis reviewed CT images from 40 patients with Bouveret’s syndrome and discovered pneumobilia in 60%, ectopic gallstones in 42%, and signs of gastric or duodenal obstruction in 33%, respectively [12]. In our case, Rigler’s triad was not visible on a conventional X-ray, but multi-detected CT (MDCT) revealed a distended stomach, an ectopic gallstone lodged in the third portion of the duodenum, pneumobilia, and cholecystoduodenal fistula. Based on these findings, Bouveret’s syndrome was diagnosed.

Regarding the treatment strategy for Bouveret’s syndrome, whether or not cholecystectomy and fistula repair following stone extraction should be performed in the same operation as one-stage surgery remains debatable [5-8]. It has been reported that if the fistula remains, there is a risk of biliary complications such as cholecystitis and retrograde cholangitis, recurrence of gallstone ileus, and an increased risk of gallbladder carcinoma [4,13]. On the other hand, when the cystic duct is patent and there are no residual stones in the gallbladder, most cholecystoduodenal fistulas close spontaneously [3]. When a fistula is left untreated, the recurrence rate of gallstone ileus is less than 5%, and although there is a relationship between fistulas and gallbladder carcinoma, the risk of developing gallbladder carcinoma is modest [8]. As one-stage surgery is considered invasive, in patients with a critical condition, advanced age, and serious comorbidities, or in cases where the local inflammation is so severe that it makes the surgery very challenging and increases the risk of intraoperative complications, a two-stage surgery consisting of an urgent stone removal with subsequent elective cholecystectomy and fistula repair after the inflammation around the gallbladder has improved is considered [4].

Despite the progress in medical science, Bouveret’s syndrome still has a high mortality rate, attributed mainly to the characteristics of the afflicted patients, who are of advanced age, have multiple comorbidities, and have complicated cases with severe inflammation [4]. In the current case, we dissected the gallbladder from its fundus and performed a subtotal cholecystectomy. The fundus first technique and a subtotal cholecystectomy should be considered, especially in cases where the inflammation surrounding the cholecystoduodenal fistula is so extensive. In addition to these surgical procedures, intraoperative cholangiography is a valid method to identify the cystic duct and its orifice with certainty. We elevated the resected jejunum on the anorectal side retrocolically and repaired the duodenal defect using the jejunal patch with an all-layer interrupted suture. Even in this complicated case with severe inflammation, the bailout procedure was effective, and we safely performed one-stage surgery without causing any bile duct injury or stenosis.

## Conclusions

We present a case of Bouveret’s syndrome, in which the operation was performed as a one-stage surgery. When general conditions allow for a surgical procedure, it is important to take the patient’s condition and severity into consideration for the development of an appropriate surgical strategy. Like the current case, one-stage surgery is feasible for Bouveret’s syndrome by surgical techniques such as the bailout procedure of the inflamed gall bladder and repairing the fistula using a jejunal patch.
